# Identification of genes associated with blood feeding in the cat flea, *Ctenocephalides felis*

**DOI:** 10.1186/s13071-015-0972-5

**Published:** 2015-07-14

**Authors:** Wayne K. Greene, Marion G. Macnish, Kim L. Rice, R.C. Andrew Thompson

**Affiliations:** School of Veterinary and Life Sciences, Murdoch University, Perth, W.A. 6150 Australia; Present address: INSERM UMR 944, Equipe Labellisée par la Ligue Nationale contre le Cancer, Institut Universitaire d’Hématologie, Hôpital St. Louis, Paris, France

**Keywords:** Cat flea, *Ctenocephalides felis*, Blood feeding, cDNA, Suppression subtractive hybridization

## Abstract

**Background:**

The cat flea (*Ctenocephalides felis)* is a blood-feeding ectoparasitic insect and particular nuisance pest of companion animals worldwide. Identification of genes that are differentially expressed in response to feeding is important for understanding flea biology and discovering targets for their control.

**Methods:**

*C. felis* fleas were maintained and fed for 24 h using an artificial rearing system. The technique of suppression subtractive hybridization was employed to screen for mRNAs specifically expressed in fed fleas.

**Results:**

We characterized nine distinct full-length flea transcripts that exhibited modulated or *de novo* expression during feeding. Among the predicted protein sequences were two serine proteases, a serine protease inhibitor, two mucin-like molecules, a DNA topoisomerase, an enzyme associated with GPI-mediated cell membrane attachment of proteins and a component of the insect innate immune response.

**Conclusions:**

Our results provide a molecular insight into the physiology of flea feeding. The protein products of the genes identified may play important roles during flea feeding in terms of blood meal digestion, cellular growth/repair and protection from feeding-associated stresses.

## Background

Arthropod ectoparasites represent a considerable nuisance and cause of suffering and economic losses in companion animals, livestock and humans. Among these, the cat flea *Ctenocephalides felis* is a widespread pest across temperate and tropical areas of the world with particular importance in domestic pets [1]. *C. felis* is the dominant flea species infesting both dogs and cats, and as an obligate hematophagous parasite is additionally capable of causing harm by acting as a disease vector [2]. This includes transmission of the bacterial diseases flea-borne spotted fever (*Rickettsia felis*) and cat-scratch disease (*Bartonella henselae*), and being an intermediate host of the intestinal cestode *Dipylidium caninum*, which is spread to dogs and cats via ingestion of infected fleas. Besides its roles in the spread of infectious disease, *C. felis* is also notable for causing pruritic skin disease (flea allergy dermatitis) in animals, particularly dogs, which are immunologically hypersensitive to flea bites [3].

Current control measures for *C. felis* are based on chemical agents, which may have complementary activities such as insecticides and insect growth regulators [4]. Further improvements in flea control will be greatly aided by detailed knowledge of flea physiology at a molecular level. In the case of the cat flea, this knowledge remains fragmentary. Identification of genes expressed in the flea in response to feeding is of key importance for elucidation of the mechanisms that permit successful feeding, digestion, immune defence and reproduction. Several feeding-specific genes have been isolated from the flea to date, including those encoding digestive proteases, protease inhibitors and synaptic vesicle proteins [5–7], however many other components involved in the physiology of feeding remain to be identified.

In this study we used an untargeted approach of suppression subtractive hybridization [8], coupled to PCR verification assays to identify *C. felis* genes differentially expressed upon feeding. A total of nine full-length cDNA sequences were obtained which showed homology to proteins associated with digestion, defence and cell proliferation. Expanded knowledge of how flea species respond to the physical, chemical and biotic stresses of blood feeding may facilitate new approaches for flea control through the exploitation of novel molecular targets.

## Methods

### Flea rearing

A colony of cat fleas (*Ctenocephalides felis felis*) was maintained using an artificial rearing system [9] consisting of a plexiglas temperature-controlled chamber (Artificial Dog, Flea Data Inc. Freeville, NY, USA). Within this apparatus, fleas were contained in screened vessels and fed on citrated bovine blood through a Parafilm membrane (Bemis Company Inc, Oshkosh, WI, USA). To complete the lifecycle, eggs were removed from the feeding vessels 2–3 times weekly and flea larvae reared on a mixture of pine sawdust, flea faeces, ground guinea pig pellets and powdered brewer’s yeast. Resultant cocoons were retrieved and stored in sealed containers for adult emergence. For the fed and unfed comparison, fleas were allowed to feed for 24 h while unfed fleas were never exposed to a blood meal. Adult fleas were stored at −80 °C in batches of approximately 100. Midgut and carcass tissues were obtained by dissecting pre-chilled adult fleas in PBS with fine forceps under a stereomicroscope using a cold light source.

### RNA Isolation and cDNA Synthesis

Total RNA was extracted from whole fleas or flea tissues in Trizol reagent (Life Technologies, Carlsbad, CA, USA) using a homogenizer (DIAX 600, Heidolph, Schwabach, Germany) and purified following the manufacturer’s protocol. Poly A+ mRNA was enriched from total RNA by oligo-dT cellulose chromatography (Life Technologies). For first strand cDNA synthesis, Poly A+ RNA (5 ug) was reverse transcribed with oligo(dT)20 using Thermoscript (Life Technologies) at 60 °C for 80 min. Second-strand cDNA synthesis was performed in second-strand synthesis buffer (20 mM Tris–HCl [pH 7.4], 100 mM KCl, 5 mM MgCl_2_, 10 mM (NH_4_)_2_SO_4_, 10 mM DTT, 50 μg/ml BSA), with 150 μM beta-NAD, 5 U of *E. coli* DNA ligase (New England Biolabs, Ipswich, MA, USA), 3 U of RNase H (Life Technologies), and 40 U of *E. coli* DNA polymerase I (Life Technologies) in a final volume of 200 ul and incubating the mixture at 15 °C for 2 h and then at 22 °C for 1 h. Double-stranded cDNA was phenol/chloroform extracted, ethanol precipitated in the presence of 4 μg of glycogen carrier, and resuspended in TE buffer (10 mM Tris–HCl [pH 7.4], 1 mM EDTA).

### Suppression subtractive hybridization (SSH)

Selective amplification of flea genes differentially expressed in response to feeding was achieved using the PCR-Select cDNA Subtraction Kit (Clontech Laboratories, Mountain View, CA, USA) according to the manufacturer’s instructions. Briefly, double-stranded cDNA from both fed and unfed whole fleas was fragmented by Rsa I digestion. Subtraction was achieved by hybridizing adapter-ligated cDNA from fed fleas as the tester in the presence of an excess of unfed flea cDNA as the driver. Differentially expressed cDNAs were PCR amplified with Advantage PCR polymerase mix (Clontech) and cloned using the pCR2.1 T/A cloning kit (Life Technologies). Two hundred clones were isolated from the subtracted library in 96-well plates containing LB medium supplemented with ampicillin and grown overnight. An aliquot from each culture was gridded onto LB-ampicillin agar plates, grown overnight at 37 °C and then transferred onto nylon membranes (Magna, ThermoFisher Scientific, Waltham, MA, USA). The filters were denatured for 5 min with 0.5 M NaOH, 1.5 M NaCl, neutralized for 5 min with 0.5 M Tris–HCl (pH 7.4), 1.5 M NaCl and then screened with probes labelled with ^32^P using the Rediprime system (GE Healthcare, Piscataway, NJ, USA). Probes were generated from 50 ng of each of fed and unfed unsubtracted cDNA in order to identify clones of differentially expressed genes. Plasmids were purified from clones showing at least two-fold higher hybridization to the labeled fed cDNA using a Mini Kit system (Qiagen, Hilden, Germany). Automated cycle sequencing of plasmid DNA inserts was performed with an ABI PRISM Model 377 DNA Sequencer (Applied Biosystems, Foster City, CA, USA) using the PRISM BigDye Terminator Cycle Sequencing Kit (Applied Biosystems).

### Rapid amplification of cDNA 5’ and 3’ ends (RACE)

The 5’ and 3’ ends of differentially expressed cDNAs were amplified by RNA ligase-mediated RACE using the GeneRacer system (Life Technologies) according to the manufacturer’s instructions. Briefly, 5 μg of total RNA extracted from fed fleas was treated with calf intestinal phosphatase and tobacco acid pyrophosphatase to allow subsequent ligation of mRNA, but not truncated and non-mRNA species, to the GeneRacer RNA oligonucleotide adapter. Reverse transcription of adapter-ligated mRNAs was performed in 20 μl reaction volumes using Thermoscript primed with the GeneRacer Oligo dT primer. Aliquots (2 μl) of the resultant cDNA containing full-length 5’ or 3’ ends were then used as a template for nested PCR using gene-specific primers (Table 1). The PCR amplifications were performed with *Tth* Plus DNA polymerase (Fisher Biotec, Perth, W.A., Australia) in 50 μl reaction volumes using an automated thermal cycler (PTC-100, MJ Research, Waltham, MA, USA). A 30-cycle touchdown protocol was employed with an initial annealing temperature of 63 °C for 1 min, decreasing by 1 °C every second cycle down to 55 °C for the final 14 cycles. Denaturation was carried out at 97 °C for 30 s and extension at 72 °C for 1 min 30 s. For the second (nested) PCR, 1 μl of the initial reaction was used as template DNA. PCR products were visualized on ethidium bromide-stained agarose gels, then cloned into the pCR 2.1 TA cloning vector and sequenced. The BLAST program was used to search for homologous genes and for multiple sequence alignments. Analysis of secretory signal peptide sequences was performed using SignalP 4.1 program [10].

**Table 1:** Primer sequences used for RT-PCR and RACE

Transcript ID		
RT-PCR	Forward Primer	Reverse Primer
B2	tgctctcatcaaagtttctagtgc	ccagacgaaagacgtgacaccaac
S5	acccagtggctcgctccgcttatg	gctaacataggcagacaagccac
S16	acgacgtcgaacgttttgtgatgc	gccttgcaaatttcaccaccct
B43	aacctaaatctgatggcagtgatg	cacaattttgtatctgagctttcc
S49	actgttctatccctggtgtcaatg	gacaagaaccattcttgaatcctg
B52	catgggtggaatgatattggttac	gttgcctaataaatgctgtgtcag
S58	ccatctgtagcctacgactatgtc	agcgctcacgtagtcagcaacaa
S61	atgcacatatcccaatatggatac	gtttcctaagaacacctttgcaa
B68	agtgaccaccacttcctatgcaac	gtaactggagtggaaacaacattg
RACE	5’ RACE Primer	3’ RACE Primer
B2	gcactagaaactttgatgagagca	gttggtgtcacgtctttcgtctgg
S5	cataagcggagcgagccactgggt	gtggcttgtctgcctatgttagc
S16	gcatcacaaaacgttcgacgtcgt	agggtggtgaaatttgcaaggc
B43	catcactgccatcagatttaggtt	ggaaagctcagatacaaaattgtg
S49	cattgacaccagggatagaacagt	caggattcaagaatggttcttgtc
B52	gtaaccaatatcattccacccatg	ctgacacagcatttattaggcaac
S58	gacatagtcgtaggctacagatgg	ttgttgctgactacgtgagcgct
S61	gtatccatattgggatatgtgcat	ttgcaaaggtgttcttaggaaac
B68	gttgcataggaagtggtggtcact	caatgttgtttccactccagttac

### Reverse transcriptase PCR (RT-PCR) analysis

For cDNA synthesis, whole flea, flea gut or flea carcass total RNA (5 μg) from 24 h fed or unfed fleas, was treated with DNAse I enzyme (DNA-*free*, Life Technologies) and reverse transcribed with oligo (dT) using Thermoscript according to the manufacturer’s instructions. PCR amplifications were performed with *Tth* Plus DNA polymerase (Fisher Biotec) in 25 μl reaction volumes using the 30-cycle touchdown protocol as per RACE. The primer sequences used are shown in Table 1. PCR products were visualized on ethidium bromide-stained agarose gels.

## Results

### Identification of genes associated with blood feeding in cat fleas by suppression subtractive hybridization (SSH)

**Fig. 1:**
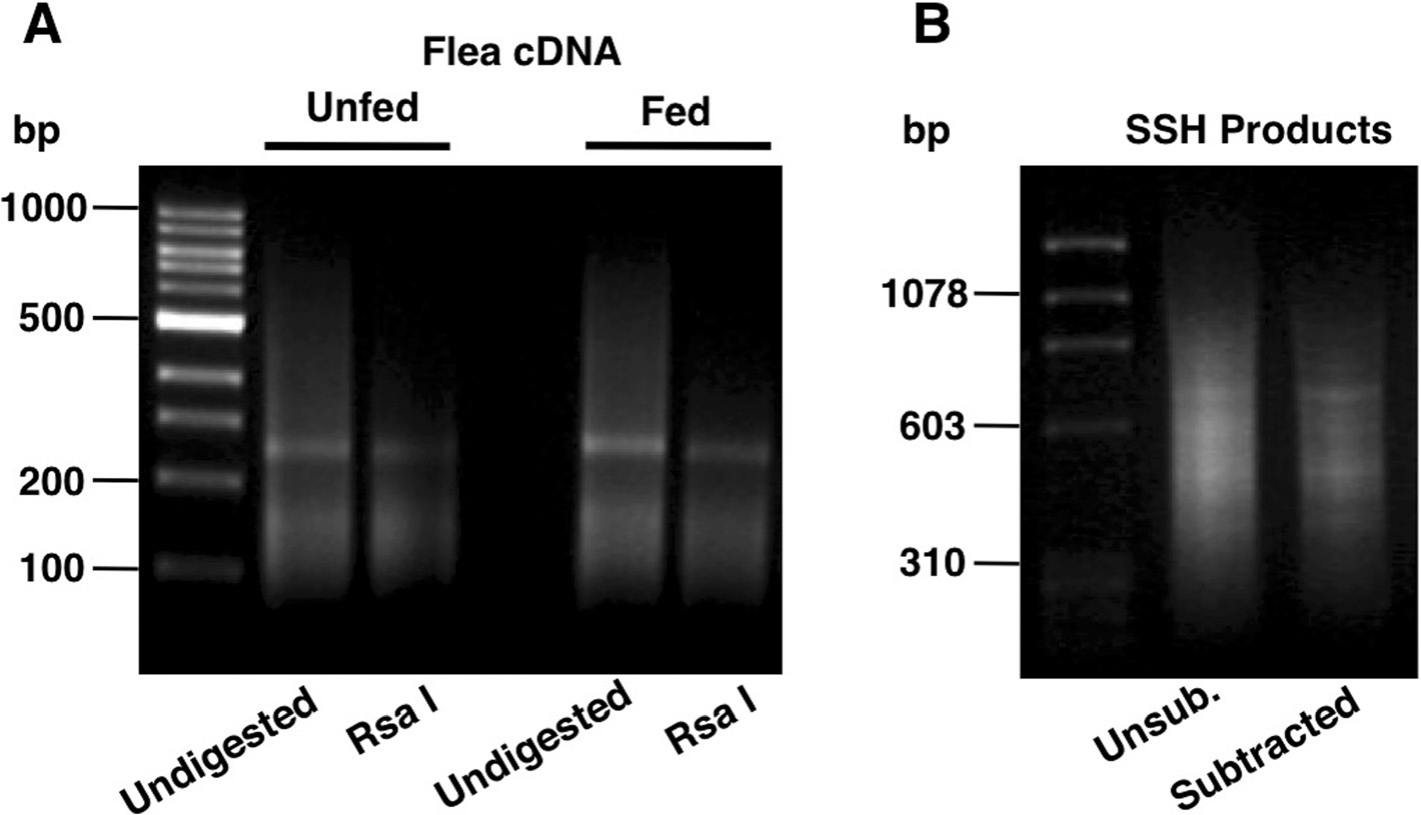
**a** Agarose gel electrophoresis showing double-stranded cDNA generated from fed and unfed *Ctenocephalides felis* fleas before and after Rsa I fragmentation. **b** Enrichment of cDNA products differentially associated with feeding by suppression subtractive hybridization (SSH). Subtraction was achieved by hybridizing adapter-ligated cDNA from fed fleas as the tester in the presence of an excess of unfed flea cDNA as driver. Unsubtracted fed flea cDNA products are shown for comparison

**Fig. 2:**
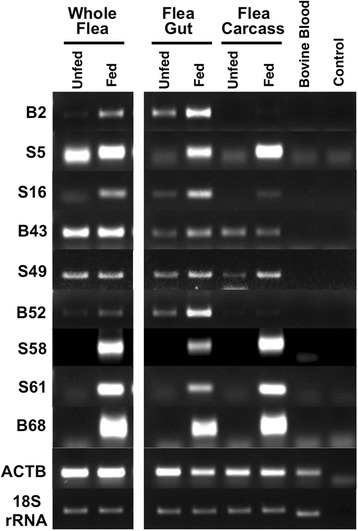
Differential gene expression between unfed and fed cat fleas (*Ctenocephalides felis*). cDNA from whole fleas, flea gut or flea carcass harvested before, or 24 h after a blood meal were assessed by semi-quantitative RT-PCR. Beta-actin and 18S RNA were amplified as controls for the amount of cDNA used in each reaction. cDNA from bovine blood used for flea feeding and a no-template reaction were included as negative controls. The results shown are representative of two independent experiments

To obtain cloned sequences of transcripts differentially expressed in fed fleas, polyA+ RNAs from whole cat fleas before and 24 h after a blood meal were converted to cDNA, fragmented with Rsa I and compared by the SSH procedure (Fig. 1). SSH was performed with fed flea cDNA acting as the tester in the presence of an excess of unfed flea cDNA as the driver. This was designed to identify transcripts that are over-expressed or up-regulated in response to feeding. Enriched SSH bands detected after agarose gel electrophoresis (Fig. 1b), were cloned and 200 randomly selected recombinant colonies subjected to sequential hybridization with unsubtracted cDNA from fed and unfed fleas as probes in order to confirm differential expression. Preliminary sequence analysis of 75 clones showing at least two-fold higher hybridization to fed flea cDNA, revealed a majority to be novel, low quality or redundant sequences. Nine candidates (B2, S5, S16, B43, S49, B52, S58, S61, B68), confirmed to be independent transcripts, were selected for full-length cloning and sequence analysis following 5’ and 3’ RACE. The GenBank accession numbers for these nucleotide sequences are KR534879 to KR534887, respectively. Differential expression of these 9 transcripts were examined in more detail and with greater sensitivity by semi-quantitative RT-PCR using RNA generated from fed (24 h) and unfed whole flea, midgut and carcass. As shown in Fig. 2, a majority of the transcripts (6/9) showed higher expression in both flea midgut and carcass, while two (B2 and B52) showed differential expression that was restricted to flea gut only. A final transcript (B43) appeared to show higher expression in fed midgut but lower expression in fed carcass. Thus, all 9 fully characterised mRNA sequences were confirmed to be differentially expressed in fed flea tissues and appear to be modulated by blood feeding.

### Characterization of feeding-associated transcripts

**Fig. 3:**
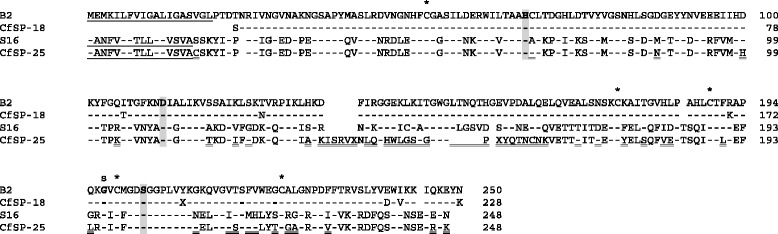
Alignment of the predicted amino acid sequences of four related *C. felis* chymotrypsin-like serine protease genes. Residues identical to sequence B2 are shown as dashes. The predicted N-terminal signal peptide for secretion is underlined. The catalytic triad residues are in bold and shaded. Conserved cysteines are marked with an asterix and the primary substrate determinant residue with an ‘s’. Residues differing between S16 and CfSP-25 are double-underlined

The nine sequences were subjected to a homology search and almost all (8/9) had homologous sequences in the nr-protein database and thus could have their functions predicted (Table 2). One short transcript of 0.6 kb (S5) encoding a small protein of 111 amino acids had no significant similarity to sequences in the database and therefore its potential function is unknown. Among the annotated gene products, most (6/8) possessed a predicted signal peptide and therefore likely represent extracellular proteins. These could be classified into two broad classes. Two were digestive enzymes (B2 and S16) with very high similarity (97 % and 78 %, respectively) with previously described *C. felis* chymotrypsin-like serine proteases CfSP18 and CfSP25 [5]. An alignment of the predicted amino acid sequences of these serine proteases is shown in Fig. 3. The remainder appeared to be molecules with various protective roles, namely a serine protease inhibitor (serpin; S49) with high similarity (96 %) to a previously described *C. felis* serpin [6], a novel peptidoglycan recognition protein (PGRP; B52) with 61 % homology to a *C. felis* PGRP LB-like protein [11], and two related novel mucins (S58 and B68), both with 36 % homology to a predicted Japanese medaka mucin-17-like protein [12]. The remaining two annotated gene products lacked predicted signal peptides and therefore likely represent intracellular proteins. One (B43) showed significant (38 %) amino acid homology with red flour beetle topoisomerase II [13], an enzyme associated with DNA replication. The other (S61) was 47 % homologous to diamondback moth glycophosphatidylinositol (GPI) mannosyltransferase 2, an enzyme involved in the biosynthesis of the GPI anchor for the membrane attachment of GPI-anchored proteins.

**Table 2:** Sequence characteristics of *C. felis* gene transcripts associated with feeding

Transcript ID	Full Transcript Size (kb)	Predicted Size of Encoded Protein (aa)	Signal Peptide	Genbank Homology Result (Best Match)	% aa Identity	Accession N^o^	E Value	Biological Function
B2	0.9	250	Y	Chymotrypsin-like serine protease CfSP-18 (*Ctenocephalides felis*)	97	AF053907.1	6e-163	Proteolytic digestive enzyme
S5	0.6	111	N	No significant similarity found	-	-	-	-
S16	0.9	248	Y	Chymotrypsin-like serine protease CfSP-25 (*Ctenocephalides felis*)	78	AF053912.1	7e-138	Proteolytic digestive enzyme
B43	1.2	242	N	DNA topoisomerase II (*Tribolium castaneum*)	38	EFA01344.1	9e-06	DNA replication
S49	1.8	405	Y	Serine protease inhibitor (Serpin) 3 (*Ctenocephalides felis*)	96	AY150534.1	0.0	Serine protease regulator
B52	1.0	201	Y	Peptidoglycan recognition protein LB-like (*Ctenocephalides felis*)	61	GU059275.1	5e-88	Innate immunity pattern recognition receptor
S58	1.2	329	Y	Mucin-17-like (*Oryzias latipes*)	36	XP_011487420.1	6e-10	Protection and lubrication of epithelial linings
S61	2.8	468	N	GPI mannosyltransferase 2 (*Plutella xylostella*)	47	XP_011549227.1	1e-141	GPI-mediated membrane attachment of GPI-anchored proteins
B68	1.3	344	Y	Mucin-17-like (*Oryzias latipes*)	36	XP_011487420.1	12e-09	Protection and lubrication of epithelial linings

## Discussion

The purpose of this study was to provide insight into gene expression changes associated with blood meal processing in the cat flea *C. felis*. To this end, suppression subtractive hybridization was used to identify a diverse group of nine genes that were up-regulated, or exclusively expressed, in the cat flea *C. felis* during a 24 h period of feeding. Genes associated with feeding included those encoding proteolytic digestive enzymes as well as those encoding several proteins putatively involved in a variety of protective roles. Most of these sequences had a predicted signal peptide for extracellular secretion. These findings were broadly consistent with other transcriptomic studies demonstrating differential gene expression in response to blood feeding in fleas and other hematophagous ectoparasites [5, 6, 11, 14–16].

Two of the identified feeding-associated genes (B2 and S16) were serine proteases. Like many other blood-feeding insect species, *C. felis* possesses a large number of serine protease genes [5] that are involved in a range of biological processes including digestion, zymogen activation and immune defence. Indeed, they constitute the most abundant digestive enzymes within the midgut owing to the protein-rich nature of the blood meal. Their functional importance means that they may have application as targets for insect and ectoparasite control [17–20]. Transcript B2 was 97 % homologous at the protein level with a previously identified *C. felis* chymotrypsin-like serine protease, CfSP-18 [5], thus B2 and CfSP-18 appear to be the same gene. By contrast, S16 appears to be a previously undiscovered serine protease, as it possessed only 78 % amino acid sequence homology with its best match, a *C. felis* chymotrypsin-like serine protease CfSP-25 [5]. The upregulation of serine protease enzymes for blood digestion has been well documented in various hematophagous arthropods [15, 21–24]. Functionally, B2 is likely to have a specific role in blood meal digestion since it was exclusively expressed in flea midgut and upregulated upon feeding. S16 was additionally expressed in flea carcass after feeding, indicating that it may have other roles besides digestion.

One identified feeding-associated gene (S49) belonged to the ubiquitous serine protease inhibitor (serpin) superfamily, which plays crucial roles in the regulation of many physiological processes by limiting the activity of proteases [25]. Not surprisingly, serpins have been explored as potential targets for the control of insects and ectoparasites [26–29]. *C. felis* has previously been shown to possess multiple serpins [6], one of which (serpin 3) was 96 % homologous with S49, thus S49 may be equivalent to *C. felis* serpin 3. It may have a role in feeding before and after ingestion of a blood meal by regulating the activity of digestive proteases and protection of the gut from deleterious proteolytic activity. S49 was expressed in both flea midgut and carcass and this was increased in both tissues in response to feeding. Thus, it would also appear to be involved in processes other than blood meal digestion. Serpins have been implicated in numerous processes in insects including reproduction, development, preventing activation of blood clotting, longevity and innate immunity [25].

Alterations in the expression of genes with an immune function have been observed after a blood meal in various arthropod ectoparasites [14, 30–33], which possess a range of molecules for innate protection against microorganisms ingested during feeding. In this study, B52 was identified as a novel gene whose encoded product has 61 % homology to a paralogous *C. felis* LB-like peptidoglycan recognition protein (PGRP) [11]. Insect PGRP-LB proteins have catalytic activity for digestion of peptidoglycan and their function may be to modulate activation of the insect immune system by the bacterial cell wall [34]. In this way, the intensity of the insect immune response is tightly adjusted to levels of ingested bacteria. In keeping with such a role, B52 expression in the flea was largely confined to the midgut.

Two further putative extracellular proteins identified (S58 and B68) were novel mucins previously undescribed in the flea. Expression of S58 and B68 was completely absent in unfed fleas, but each was transcriptionally activated in midgut and carcass in response to feeding. No significant homology was found with any known insect proteins. However, both S58 and B68 showed 36 % homology to their best match, a Japanese medaka mucin-17-like protein and were predicted to be heavily O-glycosylated. The digestive tract of the adult flea is unusual in that it reportedly lacks a protective peritrophic membrane commonly seen in other insects [35]. It is therefore plausible that such mucin-type glycoproteins function as an inducible physicochemical barrier to protect midgut and other epithelia from attack by digestive enzymes or from interaction with microbial pathogens and their toxins. Mucins, which might also provide protection from chemical and physical stresses of the blood meal itself, may have practical utility for the biocontrol of arthropod pests [36–38].

Two additional genes were identified that were not secretory, namely B43 and S61, and these appear to encode a topoisomerase II and a glycophosphatidylinositol (GPI) mannosyltransferase 2 enzyme, respectively. B43 expression increased following a blood meal specifically in the midgut and, given the function of topoisomerases in DNA replication, may reflect enhanced cell division in this organ after feeding. In support of this hypothesis, evidence from the fruit fly (*Drosophila melanogaster*) indicates that the insect midgut is a dynamic organ that can undergo growth in response to feeding leading to a net increase in intestinal cells [39]. Moreover, the intake of a large blood meal, whilst essential for haematophagous insects, creates stresses that may induce cell death [40]. Thus, cell proliferation may additionally be required in order to repair and regenerate the midgut epithelium. The expression of S61, which also encoded an intracellular enzyme, was exclusively detectable in fed fleas, both in midgut and carcass tissue. As a putative GPI mannosyltransferase 2, S61 is likely to function in the biosynthesis of GPI anchors, which are critical for cell membrane attachment of a diverse range of extracellular proteins via their carboxyl termini. These include specific cell adhesion molecules, cellular receptors, hydrolytic enzymes and regulatory proteins [41]. The transcriptional activation of a GPI mannosyltransferase 2 gene points to a potential plethora of cell surface changes in the flea in response to feeding, involving increased GPI-anchoring of proteins in various tissues, including the midgut.

## Conclusions

The identification of genes whose expression is induced by blood feeding is a key step in understanding flea physiology and flea interactions with their hosts, as well as the transmission of flea-borne pathogens. Such molecules may also have practical utility, representing potential targets for the development of new means of controlling fleas via either chemical or immunological approaches. Further studies utilizing higher throughput technologies are warranted in order to generate a comprehensive picture of gene expression changes in the flea in response to feeding, which may ultimately open new avenues for dealing with this ubiquitous parasite and pest.
